# Oral health-related quality of life in patients with upper gastrointestinal and hepatic disorders in Pakistan: validation of the Oral Health Impact Profile-14 in the Urdu language

**DOI:** 10.1038/s41405-018-0002-8

**Published:** 2018-04-27

**Authors:** Ibrahim Warsi, Anjum Younus, Abdur Rasheed, Javeria Ahmed, Hafsa Mahida, Rimsha Hashmi, Ambrina Qureshi

**Affiliations:** 1 Dr. Ishrat-ul-Ebad Khan Institute of Oral Health Sciences, Dow University of Health Sciences, Karachi, Pakistan; 2 Department of Community Dentistry, Dr. Ishrat-ul-Ebad Khan Institute of Oral Health Sciences, Dow University of Health Sciences, Karachi, Pakistan; 3 Department of Research, Dow University of Health Sciences, Karachi, Pakistan; 4 Ziauddin College of Dentistry, Ziauddin University Hospital, Karachi, Pakistan; 5 Department of Operative Dentistry, Rehmat Memorial Dental Hospital, Abbottabad, Pakistan

## Abstract

**Introduction:**

The Oral Health Impact Profile (OHIP-14) has been used extensively to measure the impact of oral disease on oral health-related quality of life (HRQoL) but has not been validated in the Urdu language or tested in gastroenterology.

**Aims:**

To validate the OHIP-14 for use in Pakistan and its ability to assess oral health in patients with upper gastrointestinal (GI) and hepatic disorders.

**Design:**

Multicentre, cross-sectional.

**Setting:**

Four major tertiary care hospitals.

**Methods:**

The OHIP-14 was tested for reliability and validity in 700 patients referred for oesophago-gastro-duodenoscopic (OGD) investigation of the symptoms of upper GI or hepatic disease. Socio-demographic details and oral examination findings (for oral lesions and DMFT) were recorded.

**Results:**

The mean (±standard deviation) total OHIP-14 score (range 0–56) was estimated to be 23.38 ± 10.47, indicating a significant impact of upper gastrointestinal and hepatic disorders on oral health. The reliability coefficient of the OHIP-14 was above 0.7 threshold, and the tool had good internal consistency (*α* = 0.83). When associated with worsening DMFT (decayed, missed, and filled teeth) index value, the highest correlations (*p* < 0.01) were detected with functional limitation (rs = 0.234), physical disability (rs = 0.230), and psychological discomfort (rs = 0.221).

**Conclusion:**

The OHIP-14 is a precise and valid instrument for assessing oral-HRQoL in a gastroenterological setting amongst Pakistani population.

## INTRODUCTION

Oral health is a state of being free from mouth and facial pain, oral infection and sores, periodontal disease, tooth decay, tooth loss, oral or throat cancer, and other conditions that limit the ability to bite, chew, smile, or speak, and negatively impact psychosocial well-being.^1,2^ Health-related quality of life (HRQoL) instruments are based on a conceptual framework that predicts daily functioning and well-being based on subjective experiences of physical, social, and emotional health.^3^

The Oral Health Impact Profile (OHIP) measures oral health and its impact on quality of life.^4^ The original version contained 49 items, but has been shortened to the OHIP-14,^5^ which contains 14 items and evaluates oral health on the basis of functional limitation, physical pain, psychological discomfort, physical disability, psychological disability, social disability, and handicap.^6^ This modified version has demonstrated consistency, responsiveness to change, and adequate cross-cultural reliability.^7–9^

Oral health is significantly compromised due to gastroenterological (inflammatory, ulcerative, neoplastic, and reflux diseases) and hepatic (commonly, hepatitis and chronic liver disease) illnesses; which leads to an array of oral manifestations such as oral ulceration, burning-mouth syndrome, tooth erosion, halitosis, xerostomia, dysphagia, dysgeusia, growth of opportunistic infections, gingival and periodontal disease, and various other hard/soft-tissue pathologies.^10–14^ Direct contact of regurgitated gastric acid is mainly involved in dental erosions in patients with gastroesophageal reflux disease (GERD). A hospital-based study showed that among patients with frequent reflux symptoms, dental erosions were reported in 64.5% as compared to controls.^15^ Another study suggests that oral health indices relevant to periodontitis may play a role in the aetiology of gastric precancerous lesions and gastric cancer.^16^ Studies measuring HRQoL in a gastroenterological setting are lacklustre and to our knowledge, this was the pioneer in assessing oral HRQoL (OHRQoL) in this disease-specific population.^17,18^

Oral health awareness is poor in Pakistan, mainly because of lack of education.^1,2,19^ There is an urgent need for awareness among Pakistanis that poor oral health is associated with poor general health. Many systemic diseases either originate in or can be screened for at an early stage in the oral cavity, hence the emergence in dentistry of the concept of screening for systemic disease and/or treating oral morbidity as a manifestation of systemic disease.^10^

The OHIP-14 has been translated, validated, and implemented in many countries and languages, including Greek,^20^ Arabic,^21^ Hindi,^22^ Brazilian,^23^ Spanish,^5,24^ Sinhelese,^25^ and Burmese.^26^ However, it has not been validated in the Urdu language in Pakistan or in patients with upper gastrointestinal (GI) or hepatic disease.^17,27–31^ The aims of this study were to assess the reliability and validity of the OHIP-14 in the Pakistani population and to demonstrate its psychometric properties with regard to OHRQoL in patients undergoing endoscopy (oesophago-gastro-duodenoscopic (OGD)) for upper GI or hepatic disease.

## Materials and methods

### Design

A cross-sectional study was carried out in patients referred for OGD investigation of symptoms of upper GI or hepatic disease in Karachi and the twin cities of Rawalpindi–Islamabad, where more than 10% of the Pakistani population resides,^32^ between December 2014 and April 2015. The sampling units comprised four major tertiary care hospitals (Civil Hospital Karachi, Jinnah Postgraduate Medical Centre, Pakistan Institute of Medical Sciences Islamabad, and Holy Family Hospital Rawalpindi). A free dental consultation was provided for all study participants and intraoral examination was performed by a calibrated single examiner (IW).^33^ All participants also attended an interview during which a self-administered questionnaire was completed. Patients who had extremely limited mouth opening that precluded an oral examination, those undergoing emergency procedures, those who were unconscious or non-cooperative, and those who were attending only for follow-up were excluded.

The study protocol was approved by the ethical committees at each participating hospital before any patients were enrolled. Written informed consent was taken from all study participants after explaining the study protocol.

### HRQoL assessment using the OHIP-14

The OHIP-14 was piloted after translation into Urdu using the translation technique described by Guillemin et al.^34^ and then assessed for its reliability and validity in the Pakistani setting. A pilot study was undertaken in 30 subjects to ensure that the proforma was comprehensive. The study participants then underwent an oral clinical examination for any abnormal hard or soft-tissue findings, which might contribute adversely to oral health and health in general.

### Sample size

The sample size was calculated using OpenEpi software taking the highest-recorded mean percentage for the OHIP-14,^5^ i.e., 80% with a 5% margin of error and a 95% confidence interval. A minimum study sample size of 246 was calculated to be necessary; however, we were able to recruit a sample of 760 patients from the four tertiary hospitals, which cater patients from all states in Pakistan.

### Data collection

The proforma consisted of three domains. The first domain was history-taking to obtain data on socio-demographics and associated risk factors. The second domain consisted of an assessment of the oral, upper GI, and hepatic disorders based on the HRQoL and was measured using a universal validated index, i.e., the OHIP-14.^6^ The third domain comprised an oral examination that recorded clinical changes in the hard and soft tissues, dental caries assessment, and the presence of oral submucosal fibrosis (OSF) using the oral screening methods recommended by the World Health Organization (WHO).^35^

### Assessment of socio-demographics and risk factors

A self-administered questionnaire was used to elicit data on age, sex, monthly income and socio-economic status, race/ethnicity, residence, dietary pattern, smoking, alcohol, tea, and oral tobacco consumption, medical history, drug history (including use of non-steroidal anti-inflammatory drugs (NSAIDs), opioid painkillers, and other over-the-counter medications), and comorbidities. Findings of OGD, laboratory investigations, or detailed upper GI examination suggestive of a current upper GI or hepatic disorder were recorded.

### Oral examination

Oral examinations were performed according to an established ethical protocol.^33^ In line with WHO community indices,^35^ all patients were screened for abnormal mucosal and/or hard or soft-tissue lesions, including gingivitis, angular cheilitis, xerostomia, dental erosions, ulceration, abscess, leukoplakia, erythroplakia, lichen planus, and candidiasis. Inter-incisal mouth opening was measured and intra-oral palpation was performed to assess fibrotic banding and stiffness of the oral mucosa for diagnosis and staging of OSF.^36^ Dental hard-tissue status was assessed using DMFT (decayed, missed, and filled teeth) index values.^35^

### Statistical analysis

The data were analyzed using SPSS version 21.0 software (SPSS Inc., Chicago, IL, USA). Descriptive statistics are presented as the mean (and standard deviation) and and frequency percentage. Associations between the study variables were assessed using non-parametric tests.

The psychometric properties of the OHIP-14 tool were analyzed using reliability and validity tests.^37^ The validation process consisted of language adaptation of the original instrument, a pilot study to eliminate confusing and conflicting parts and make it culturally adaptable for all ethnicities, and the main study to assess reliability and validity.^37,38^ The reliability of the OHIP-14 is based on internal consistency and homogeneity. Internal consistency was calculated using the standardized Cronbach’s alpha and alpha item if deleted.^20,37^ The degree of homogeneity was evaluated within seven subscales on the basis of corrected item-total correlation coefficients.^25^

Convergent validity was used as a tool to establish the validity of the OHIP-14.^37^ Based on the method of evaluation followed by the previous study,^20^ our study assumed that a higher value of DMFT (i.e., poorer dentition/oral hard tissues) would be associated with higher OHIP-14 scores and hence, with lower levels of OHRQoL.^6,20^ In addition, the statistical significance of differences in mean OHIP-14 scores and DMFT index value, OSF, and oral lesions was assessed using the Mann–Whitney *U* and Kruskal–Wallis tests.

Total OHIP-14 scores were calculated using the additive scoring method devised by Robinson et al.^39^ The scores possible on the OHIP-14 range from 0 to 56, with higher scores indicating poor oral health (lower OHRQoL) and vice versa. Roumani et al.^20^ illustrate OHIP-14 cut-off values for ‘good oral health (OH)’ with OHIP-14 score <9.33 (SD ± 6.5) and ‘poor OH’ ≥11.0 (SD ± 6.9).

## RESULTS

Sixty of the 760 patients initially considered eligible for the study were excluded because of the failure to answer all questions on the questionnaires, leaving data on 700 patients available for analysis (response rate 100%, while generating a completion rate of 92%).

### Demographics

Table 1 shows the socio-demographics of the 700 patients (male, 55%; mean age 45 years) who underwent OGD. Sixty-two percent were urban-dwellers and 38% were rural-dwellers. The majority were from the Punjab (44.3%) and Sindh (39.3%) regions and most (69%) of them were of very-low socio-economic status. Habitual drinking of tea was common (77.6%), along with consumption of oral tobacco (27.1%) and cigarette smoking (21.6%). Patients with pain tended to self-medicate, most often with NSAIDs (48.9%). Weight loss of 1 kg to >20 kg was reported by 71.3% of subjects; 42.4% reported weight loss <5 kg and 10.6% reported weight loss of 10–15 kg.

**Table 1:** Socio-demographics and risk factors

Demographic characteristics		Frequency (*n*)	(%)^a^
Gender	Male	385	**55**
	Female	315	45
Age groups (in years)	12–19	51	7.3
	20–30	96	13.7
	31–40	122	**17.4**
	41–50	181	**25.9**
	51–60	130	**18.6**
	61–70	93	13.3
	70 and above	25	3.6
Residence/background	Urban	434	**62**
	Rural	266	38
Province	Sindh	275	**39.3**
	Punjab	310	**44.3**
	Balochistan	19	2.7
	Khyber Pakhtunkhwa	66	9.4
	Kashmir	30	4.3
Monthly income (in Pakistani rupees, PKR) (socio-economic status, SES)	<15000 PKR (very low SES)	483	**69**
	15000–30000 PKR (low SES)	196	28
	30000–50000 PKR (moderate SES)	21	3.0
Dietary pattern^b^	Healthy diet	86	12.3
	Satisfactory diet	312	**44.6**
	Unhealthy diet	302	43.1
Addiction profile	Smoking	151	21.6
	Alcohol	13	1.9
	Oral tobacco	190	27.1
	Tea	543	**77.6**
Comorbids	Diabetes mellitus (DM)	150	**21.4**
	Hypertension (HTN)	228	**32.6**
Pain history and NSAIDS consumption	General body pain	285	**40.7**
	(NSAIDS) Pain killer intake	342	**48.9**
Weight loss (kilograms)	0	201	28.7
	01–05	297	**42.4**
	05–10	65	9.3
	10–15	74	**10.6**
	15–20	44	6.3
	>20	19	2.7
Gastric maladies	Oesophageal varices	256	**36.6**
	GORD	187	**26.7**
	Gastritis	169	**24.1**
	Peptic ulcer disease (PUD)	58	8.3
	Oesophagitis	39	5.6
	Other minor GI disorders	129	18.4
Liver disorders	Hepatitis B	25	3.6
	Hepatitis C	230	**32.9**
	Portal gastropathy	125	**17.9**
	Chronic liver disease (CLD/DCLD)	113	16.1

*NSAIDS* non-steroidal anti-inflammatory drugs, *GORD* gastro-oesophageal reflux disease, *GI* *disorder* gastrointestinal disorders

^a^Items in bold highlight the major prevalence in each category

^b^Dietary pattern (Healthy diet refers to balanced diet; Satisfactory diet: being intermediary between healthy and unhealthy diet, specifically referring to occasionally eating junk food, while diet relatively lacks in natural abrasives like fruits/vegetables, cereals, leafy vegetables, and fibres; Unhealthy diet refers to consuming high fatty/oily meals, frequent consumption of junk food/fast food, and soft drinks, while completely devoid of natural abrasives)

Endoscopic and laboratory investigations revealed a high prevalence of hepatitis C (32.9%) and portal gastropathy (17.9%). Oesophageal varices (36.6%), GI reflux disease (26.7%), and gastritis (24.1%) were also prevalent. Diabetes (21.4%) and hypertension (32.6%) were common comorbidities.

### Distribution of OHIP-14 items

The distribution of the responses to OHIP-14 is presented in Table 2, which depicts that the majority of patients were reported as ‘occasionally having had problems’ in the last year on nine of fourteen items (ranging from life felt less satisfying (26.4%), to worsened taste (27.3%), interrupted meals (27.3%), difficulty in relaxing (29.1%), felt self-conscious (30.9%), experienced irritability (31.9%), difficulty in doing usual jobs (33.9%), unsatisfactory diet (35.6%), and having felt embarrassed (37.3%)). There were relatively fewer patients reported that they ‘often or very often’ experienced problems in the last year, except for having often felt tensed (28.4%). Mean scores ranged between 0.65 for painful aching in the mouth and 2.26 for feeling tensed because of oral problems in the past year.

**Table 2:** Distribution of OHIP-14 items, ranging from 0 (never), 1 (hardly ever), 2 (occasionally), 3 (often) to 4 (very often)

Description of item		Distribution of responses (%)^a^						
Items	Questions	0	1	2	3	4	Mean^b^	S.E
*Functional limitation*								
OH-1	Trouble pronouncing words	**66.4**	8.3	12.3	9.1	3.9	0.7571	0.0454
OH-2	Sense of taste worse	26.6	11.3	**27.3**	24.4	9.4	1.7986	0.0502
*Physical pain*								
OH-3	Painful aching in the mouth?	**65.7**	11.0	16.7	5.4	1.1	0.6529	0.0382
*Psychological discomfort*								
OH-5	Self-conscious	30.7	10.9	**30.9**	22.7	4.9	1.6014	0.0478
OH-6	Felt tense	17.1	7.1	27.7	**28.4**	19.6	**2.2614**	0.0501
*Physical disability*								
OH-4	Uncomfortable to eat	**44.0**	10.4	21.6	13.4	10.6	1.3614	0.0536
OH-7	Unsatisfactory diet	22.6	8.0	**35.6**	23.0	10.9	**1.9157**	0.0484
OH-8	Had to interrupt meals	24.7	11.1	**27.3**	24.3	12.6	1.8886	0.0512
*Psychological disability*								
OH-9	Difficult to relax	22.7	13.9	**29.1**	22.4	11.9	1.8686	0.0497
OH-10	Embarrassed	27.6	12.7	**37.3**	16.3	6.0	1.6471	0.0632
*Social disability*								
OH-11	Irritability with other people	18.4	7.7	**31.9**	29.9	12.1	**2.0957**	0.0476
OH-12	Difficulty doing your usual jobs	15.4	14.0	**33.9**	21.1	15.6	**2.0743**	0.0476
OH-13	Felt life less satisfying	17.1	17.7	**26.4**	22.4	16.3	**2.0300**	0.0498
*Handicap*								
OH-14	Totally unable to function	**34.1**	23.1	19.4	11.6	11.7	1.4357	0.0516

^a^Bold represents the major percentage prevalence reported in each of the fourteen OHIP-14 items

^b^Mean shows the average of each item response (with 0 being lowest to 4 being highest)

### Total OHIP-14 score

The total OHIP-14 score was calculated as 23.38 ± 10.47, which is much higher than the threshold for poor oral health (i.e., OHIP-14 score >11, SD ± 6.5);^20^ thus indicating a significant impact of an upper GI and hepatic disorders on oral health.

### Distribution of OHIP-14 with DMFT, OSF, and oral lesions

Table 3 illustrates the distribution of OHIP-14 mean when associated with DMFT, OSF, and oral lesions. The mean DMFT index values were categorized according to whether they were <5 or ≥5.^12,20^ The mean OHIP-14 score for a DMFT index value ≥5 was significantly higher at 26.51 ± 9.28 (*p* < 0.001). When OSF was categorized as absent, mild, moderate, or advanced, advanced OSF was associated with a significantly higher mean OHIP score of 34.84 ± 9.39 (*p* < 0.001). Statistically significant OHIP-14 scores (*p* < 0.001) were obtained for candidiasis (30.47 ± 09.71), glossitis (27.71 ± 10.06), angular cheilitis (25.96 ± 7.40), leukoplakia (24.71 ± 06.70), and ulceration (22.74 ± 9.48).

**Table 3:** Distribution of OHIP-14 with DMFT, OSF, and oral lesions

Characteristics	OHIP-14 mean (SD)/*N*	Test of significance	*p*-Value*
Number of decayed, missing, and filled teeth (DMFT)		Mann–Whitney *U*	<0.001
➢ DMFT <5	20.82 (±10.72)/384		
➢ DMFT ≥5	26.51 (±9.28)/316		
Type of oral submucosal fibrosis (OSF)		Kruskal–Wallis	<0.001
➢ No OSF	19.31 (±10.00)/308		
➢ Mild	23.82 (±8.73)/214		
➢ Moderate	28.28 (±9.40)/133		
➢ Advanced	34.84 (±9.39)/45		
Oral lesions		Kruskal–Wallis	<0.001
➢ No oral lesions	18.88 (±10.19)/224		
➢ Ulceration	22.74 (±9.48)/240		
➢ Angular cheilitis	25.96 (±7.40)/28		
➢ Glossitis	27.71 (±10.06)/108		
➢ Leukoplakia	24.71 (±06.70)/14		
➢ Candidiasis	30.47 (±09.71)/86		

*Level of significance = *p*-value < 0.01

### Reliability

The Cronbach’s alpha (*α*) coefficient for the OHIP-14 was estimated to be 0.837 (Table 4), indicating very good internal consistency.^5,20,25^ The homogeneity of the scale was evaluated on the basis of corrected item–total correlation coefficients. The degree of homogeneity within the seven subscales of the OHIP instrument ranges from 0.1 (psychological discomfort) to 0.7 (physical and psychological disability).^22^ The corrected item–total correlation coefficient values ranged from 0.335 to 0.587, i.e., >0.2, which is the standard for inclusion of an item in OHIP subscale.^22,37^

**Table 4:** Reliability analysis: corrected item-total correlation, Cronbach’s alpha*, and alpha if item deleted

Items	Corrected item-total correlation	Cronbach’s alpha if item deleted
Trouble pronouncing?	0.417	0.831
Taste worsened?	0.409	0.831
Painful aching?	0.361	0.833
Uncomfortable to eat?	0.505	0.825
Self-conscious?	0.413	0.831
Felt tense?	0.578	0.820
Diet been unsatisfactory?	0.572	0.821
Interrupt meals?	0.586	0.819
Difficult to relax?	0.461	0.828
Been embarrassed?	0.335	0.840
Irritable a bit?	0.475	0.827
Difficulty doing your usual jobs?	0.493	0.826
Life generally less satisfying?	0.587	0.820
Totally unable to function?	0.467	0.827
Mean	0.4756	0.8271

*Cronbach’s alpha value (*α* = 0.837)

### Validity

The results for the convergent validity of the OHIP-14 are shown in Table 5. According to the WHO guidelines, a DMFT index value <3 is categorized as very low caries, a score of 4–10 as average, and a score of 11–32 as poor.^35,40^ An increasing trend for OHIP-mean with worsening DMFT is evident, with most affected subscales being social disability (7.31 ± 2.63), physical disability (5.55 ± 2.73), and psychological discomfort (4.64 ± 1.89); while the least affected being physical pain (0.84 ± 1.10). All Spearman’s rank correlation coefficients were positive and statistically significant (*p* < 0.001), with the highest correlation being for the functional limitation subscale (rs = 0.234) and the lowest for the physical pain subscale (rs = 0.160).

**Table 5:** Convergent validity of OHIP-14 using decayed, missed, and filled teeth (DMFT) index

	OHIP-14 Subscales Characteristics	DMFT category			Statistics Spearman Ranks (rs) correlation^a^
		Acceptable DMFT <3 *N* = 341 % = 48.7 Mean subscale (S.D)	Average DMFT 4–10 *N* = 220 % = 31.4 Mean subscale (S.D)	Poor DMFT > 10 *N* = 139 % = 19.9 Mean subscale (S.D)	
1.	Functional Limitation	2.1056 (1.95252)	2.8545 (1.93660)	3.1871 (2.05563)	**0.234**
2.	Physical pain	0.5073 (.96289)	0.7545 (0.99484)	0.8489 (1.10934)	0.160
3.	Psychological discomfort	3.3842 (2.39034)	4.1136 (1.95633)	4.6403 (1.89968)	**0.221**
4.	Physical disability	3.8358 (2.87525)	4.7273 (2.70771)	5.5540 (2.73775)	**0.230**
5.	Psychological disability	3.1554 (2.73042)	3.6591 (1.85973)	4.1727 (1.84520)	0.220
6.	Social disability	5.6452 (3.33611)	6.3545 (2.82224)	7.3165 (2.63487)	0.194
7.	Handicap	1.2669 (1.41496)	1.4727 (1.32216)	1.7914 (1.24222)	0.172

^a^All rs values have *p*-value < 0.01; statistically significant. Bold represents the highest correlations between OHIP-14 subscales and affected DMFT

## DISCUSSION

To our knowledge, this is the first study to validate the OHIP-14 in the Pakistani population and the first to focus on OHRQoL in patients with upper GI disorders.^17,27–31^ Cross-cultural adaptation is a critical component in the validation of a health assessment tool.^5,38,41^ The convergent validity method was used to establish the validity of the OHIP-14 in our population, as described by Roumani and Montero-Martin.^5,20^ Unlike many studies,^2,5,20–25,31,42,43^ in which validity was assessed using weak tools such as oral hygiene, self-perceived status of oral health, and subjective symptoms, with comparison of both additive and simple counting; we established the validity of the OHIP-14 using a clinical parameter, i.e., effect on oral hard tissues (assessed using DMFT index), adding strength to this study. Our mean OHIP-14 scores are higher than those previously reported,^44^ and can be attributed to the underlying upper GI disease.

Our socio-demographic findings for age, sex, and socio-economic status are in agreement with those of another local study reported by Ghani et al.^29^ The literature identifies the major risk factors for upper GI disorders to be the addiction profile and dietary habits, and the confounding risk factors to be rural residence and very-low to low socio-economic status.^11^ Pakistan faces big challenges in regard to maintenance of oral health,^45^ in that the bulk of its population does not prioritize health, being addiction-prone, having neglected health issues, seeking self-medication, and often living in rural areas,^32^ with limited access to health care, all of which render individuals prone to ill health.^1,2,19^

The present study demonstrates that the presence and severity of oral lesions is associated with oral morbidity (Table 3), which compromises oral and systemic health. To date, there have been no reports of the OHIP-14 being used to address the impact of oral lesions, abnormal mucosal conditions, or OSF, so another novel element of our study is that it confirms significant associations in this regard.^5,20–25,42,43^ Oral candidiasis manifests as xerostomia, burning-mouth syndrome, altered taste sensation, redness, and soreness,^46^ and was the oral lesion with the worst impact on oral health in our study. These symptoms were severe enough to cause eating or swallowing difficulties, a diminished sense of taste, inadequate food intake, interrupted meals, and loss of appetite, culminating in weight loss in 71.3% of our study population. Weight loss is a marker of the severity and progression of disease, and oral disability might contribute to compromised health and/or decreased appetite.^41^ We observed a trend of increasing mean OHIP-14 scores with increasing severity of oral lesions. The associations between OHIP-14 scores and ulceration, angular cheilitis, glossitis, leukoplakia, and candidiasis were statistically significant. Of these, candidiasis and glossitis had the worst impact on OHRQoL.

Our data (Table 4) also indicate that the Pakistani–Urdu version of the OHIP-14 has very good internal reliability (*α* = 0.83), as found for the most recent Indian–Hindi validation study of the OHIP-14 (*α* = 0.80) by Batra et al.^22^ This can be attributed to the similarities in these regional populations, indicating that the results of these regional studies are generalizable. Similarly, *α* = 0.89 (considered excellent), as reported for the Spanish version by Montero-Martin et al.,^5^ highlights that our regional reliability *α*-values are in accordance with European studies.^5,20,22^ Internal reliability is a critical psychometric property for a health measurement tool,^37^ and our finding in this regard indicates that the OHIP-14 is suitable for the assessment of OHRQoL in the Pakistani population. In addition, our study findings revealed that OHIP-14 mean responses (ranging from 0.65 to 2.26) were relatively higher in patients suffering from oro-dental problems in the presence of systemic illness; in comparison with an Australian study,^44^ where responses of OHIP-14 were ranging from 0.14 to 1.36 and no underlying systemic illness was present that could have negatively influenced the oral health quality of the participants. Hence, this study serves as a control for comparing OHIP-14 means in healthy population versus population suffering from gastroenterological illness.

In our study, convergent validity (Table 5) pertained to two construct measures, i.e., the DMFT index and the OHIP-14, which are both theoretically and clinically related.^37^ Our present findings are consistent with those of previous validation studies, in which all researchers have agreed with the approach that functional limitation (rs = 0.234), physical disability (rs = 0.230), and psychological discomfort (rs = 0.221) have the highest correlation with compromised oral health (which in this study is measured with worsening DMFT score);^20,26,47^ and also demonstrate that the higher the DMFT index value, the poorer the oral health status, and the greater the likelihood of compromised aesthetics, speech, and mastication.^5,20–24,42,43^

More than half of our study population had a DMFT index value >3, which is beyond the acceptable threshold,^40^ and amongst that group, a significant portion (around 20%) had a value >10. These data indicate that our sample population included a substantially larger group of individuals who were at risk of caries than in previous studies, in which samples were classified as at negligible-low risk (a DMFT index value of 0.6),^26^ low–medium risk (3.2),^20^ medium risk (5.4),^48^ or high risk (8.8).^47^ Moreover, social disability, physical disability, and psychological discomfort were the items in the OHIP-14 worst affected in respect to worsening DMFT index value (i.e., poor DMFT >10, *p* < 0.01). These findings support the concept that the DMFT index has a significant role in determining oral health, and according to our data, oral health is compromised in the presence of an upper GI or hepatic disorder.^17^ In contrast, a WHO report based on Peterson’s world map of dental caries in 2003^49^ suggests that the Pakistani population is at low risk (a mean DMFT index value of <1.2 for those up to 12 years of age, and <5 for those aged 35–44 years). In light of our present findings, we would suggest that the WHO update its recent findings with regard to DMFT index values in Pakistan.^50^

Other important findings in this study were a lack of health awareness and a high rate of self-medication in Pakistanis. Low socio-economic status and poor literacy,^48^ are major drivers of health neglect in Pakistan.^1,2,19,45^ This neglect was reflected in the high proportions of subjects reporting general body pain (40%) and self-medicating with NSAIDs (48%), which are known to have adverse effects on the liver and upper GI tract.^11^ Our study has several limitations. First is its cross-sectional design, which meant that only a small window of time was available for interviews with subjects and limited the levels of association that could be found; however, this design was needed for validation of the OHIP-14. Longitudinal or case-control studies are needed in the future to detect dynamic changes in oral well-being after therapeutic interventions and to document the sensitivity and specificity of the OHIP-14 and its suitability for use in Pakistan in more detail. Second, we could not rule out the potential contributions of comorbidities and self-medication (particularly with NSAIDs) to upper GI and hepatic disease. Third, the oral lesions detected in our study population were not histologically confirmed, so our findings in this regard only have applicability for screening. It is recommended that future researchers include biopsies of oral lesions in their study protocols. Fourth, the use of DMFT index despite being widely criticized as a weak tool for distinguishing between the aetiology of decay or missing tooth, yet it is still widely used in epidemiological studies for the screening of dental decay. This study was the first in its kind to assess OHRQoL in systemic disease-specific population, therefore, DMFT was used as a central measure for the analysis of oral hard tissues. Fifth, as it was not our study objective and also by not associating between decayed, missed, and filled teeth separately, we might have missed out identifying a potential relationship with decay or missing of tooth. We strongly recommend future researchers to explore the effects on OHRQoL in systemic diseases having oral manifestations, while clinical trials and prospective longitudinal studies are needed to determine the temporal relationship between the diseases commonly encountered by gastroenterologists and dentists.

## CONCLUSION

Upper GI and hepatic disorders produce an array of oral manifestations that not only compromise oral health but also significantly affect OHRQoL. Dentists, general physicians, and gastroenterologists need to play a very proactive role in educating their patients regarding the implications of poor oral health and its effect on the quality of life. Referring to a dentist could become part of the treatment protocol in gastroenterology. The Urdu version of the OHIP-14 instrument showed similar and reliable characteristics as in the original English version and is not only validated for use in Pakistan as an oral health assessment tool in patients with upper GI or hepatic disease but can also be employed for assessment of OHRQoL among general Pakistani population with different age ranges.
